# Visfatin Induces Inflammation and Insulin Resistance via the NF-*κ*B and STAT3 Signaling Pathways in Hepatocytes

**DOI:** 10.1155/2019/4021623

**Published:** 2019-07-17

**Authors:** Yu Jung Heo, Sung-E Choi, Ja Young Jeon, Seung Jin Han, Dae Jung Kim, Yup Kang, Kwan Woo Lee, Hae Jin Kim

**Affiliations:** ^1^Department of Biomedical Sciences, Graduate School of Ajou University, Suwon, Republic of Korea; ^2^Department of Physiology, Ajou University School of Medicine, Suwon, Republic of Korea; ^3^Department of Endocrinology and Metabolism, Ajou University School of Medicine, Suwon, Republic of Korea

## Abstract

**Background:**

It has been suggested that visfatin, which is an adipocytokine, exhibits proinflammatory properties and is associated with insulin resistance. Insulin resistance and inflammation are the principal pathogeneses of nonalcoholic fatty liver disease (NAFLD), but the relationship, if any, between visfatin and NAFLD remains unclear. Here, we evaluated the effects of visfatin on hepatic inflammation and insulin resistance in HepG2 cells and examined the molecular mechanisms involved.

**Methods:**

After treatment with visfatin, the inflammatory cytokines IL-6, TNF-*α*, and IL-1*β* were assessed by real-time polymerase chain reaction (RT-PCR) and immunocytochemical staining in HepG2 cells. To investigate the effects of visfatin on insulin resistance, we evaluated insulin-signaling pathways, such as IR, IRS-1, GSK, and AKT using immunoblotting. We assessed the intracellular signaling molecules including STAT3, NF-*κ*B, IKK, p38, JNK, and ERK by western blotting. We treated HepG2 cells with both visfatin and either AG490 (a JAK2 inhibitor) or Bay 7082 (an NF-*κ*B inhibitor); we examined proinflammatory cytokine mRNA levels using RT-PCR and insulin signaling using western blotting.

**Results:**

In HepG2 cells, visfatin significantly increased the levels of proinflammatory cytokines, reduced the levels of proteins (e.g., phospho-IR, phospho-IRS-1 (Tyr612), phospho-AKT, and phospho-GSK-3*α*/*β*) involved in insulin signaling, and increased IRS-1 S307 phosphorylation compared to controls. Interestingly, visfatin increased the activities of the JAK2/STAT3 and IKK/NF-*κ*B signaling pathways but not those of the JNK, p38, and ERK pathways. Visfatin-induced inflammation and insulin resistance were regulated by JAK2/STAT3 and IKK/NF-*κ*B signaling; together with AG490 or Bay 7082, visfatin significantly reduced mRNA levels of IL-6, TNF-*α* and IL-1*β* and rescued insulin signaling.

**Conclusion:**

Visfatin induced proinflammatory cytokine production and inhibited insulin signaling via the STAT3 and NF-*κ*B pathways in HepG2 cells.

## 1. Introduction

Visfatin is an adipocytokine that is expressed predominantly in visceral adipose tissue [1] and also in skeletal muscle, bone marrow, and hepatocytes [2]. Visfatin was previously identified as a pre-B-cell colony-enhancing factor (PBEF), a cytokine expressed and secreted by lymphocytes [3], and also as nicotinamide phosphoribosyltransferase (NAMPT), an enzyme converting nicotinamide to nicotinamide mononucleotide (NMN), a precursor of nicotinamide adenine dinucleotide (NAD) [4]. Visfatin has been extensively studied in terms of its roles in obesity, insulin resistance, type 2 diabetes, and other metabolic disorders, but the findings have been contradictory [5–7].

Nonalcoholic fatty liver disease (NAFLD) is a spectrum of conditions ranging from fatty liver to nonalcoholic steatohepatitis (NASH), fibrosis, and cirrhosis [8]. Insulin resistance is considered to be the principal pathogenesis of NAFLD, and inflammation is also known to be involved in the disease progress [9]. Several adipocytokines have been suggested to play important roles in pathogenesis [10]. The role of visfatin in NAFLD has been evaluated, but the results were inconsistent [11–13], and it remains unclear whether visfatin contributes to hepatic insulin resistance and inflammation. Moreover, the effects of visfatin as an adipocytokine in hepatocytes have rarely been explored. We reported previously that visfatin stimulated gluconeogenesis in hepatocytes [14], suggesting a contribution of visfatin to hepatic insulin resistance. However, the effects of visfatin on insulin signaling in hepatocytes have not been reported previously.

Previous studies suggested that visfatin exhibited proinflammatory properties, playing roles in certain inflammatory diseases and in particular cell types [1]. Visfatin was reported to induce Interleukin-1 beta (IL-1*β*), tumor necrosis factor-*α* (TNF-*α*), and Interleukin-6 (IL-6) production by monocytes [15] and to upregulate IL-6 gene expression in human endothelial cells [16]. Another study revealed that visfatin induced migration of osteosarcoma cells and upregulation of IL-6 through nuclear factor kappa B (NF-*κ*B) [17].

Elevated levels of circulating cytokines may impair insulin signaling in peripheral organs [18]. It remains unclear how visfatin might affect liver insulin resistance and inflammation. Here, we evaluated the effects of visfatin on inflammation and insulin resistance in HepG2 cells and examined the molecular mechanisms involved.

## 2. Materials and Methods

### 2.1. Materials

Recombinant human visfatin was purchased from PeproTech (Rocky Hill, NJ, USA). The tyrosine kinase inhibitor of Janus kinase 2 (JAK2), AG490, was from Enzo Life Sciences Inc. (Farmingdale, NY, USA). The NF-*κ*B inhibitor Bay 7082 was purchased from Calbiochem (San Diego, CA, USA). Antibodies against phospho-insulin receptor (IR), total IR, insulin receptor substrate 1 (IRS-1 (Thy612)), phospho-Glycogen Synthase Kinase 3 *α*/*β* (GSK-3*α*/*β*), protein kinase B (AKT), phospho-AKT, JAK2, phospho-JAK2, signal transducer and activator of transcription 3 (STAT3), phospho-STAT3, I*κ*B kinase (IKK), phospho-IKK*α*/*β*, inhibitor of kappa B (I*κ*B*α*), NF-*κ*B, phospho-NF-*κ*B, c-Jun N-terminal kinases (JNK), phospho-JNK, and p-38 mitogen-activated protein kinases (p-38) and phospho-p-38 were purchased from Cell Signaling Technology (Danvers, MA, USA). Anti-GSK-3*α*/*β*, anti-actin, anti-phosphor-extracellular signal-regulated kinase (ERK), and anti-ERK antibodies were purchased from Santa Cruz Biotechnology Inc. (Santa Cruz, CA, USA). Anti IRS-1 (Ser307) antibody was purchased from Upstate Biotechnology Inc. (Lake Placid, NY, USA). Culture media, culture supplements, and fetal bovine serum (FBS) were obtained from Gibco-BRL (Grand Island, NY, USA).

### 2.2. Cell Culture

HepG2 cells were obtained from the American Type Culture Collection and grown in minimum essential medium (MEM) supplemented with 10% (*v*/*v*) fetal bovine serum (FBS) and antibiotics (10 *μ*g/mL streptomycin and 100 IU/mL penicillin) at 37°C in a humidified atmosphere of 95% air and 5% CO_2_ (both *v*/*v*). Before treatment with 0.1% BSA/PBS or visfatin (visfatin dissolved in 0.1% BSA/PBS), media were replaced with serum-free DMEM. To measure insulin signaling, media containing visfatin were exchanged for serum-free DMEM followed by incubation for 1 hour and then treatment with insulin for 10 minutes.

### 2.3. Immunobloting

Cells were suspended in RIPA buffer (150 mM NaCl, 1% (*v*/*v*) NP-40, 0.5% (*w*/*v*) deoxycholate, 0.1% (*w*/*v*) sodium dodecyl sulfate (SDS), 50 mM Tris-HCl (pH 7.5), and a protease inhibitor (pancreatic extract, pronase, thermolysin, chymotrypsin, and papain) cocktail (Roche Applied Science, Mannheim, Germany)) and incubated on ice for 20 min. Total proteins were extracted by differential centrifugation (13,000 × g, 10 min), and the protein concentrations in lysates were determined using a protein assay kit (Bio-Rad, Hercules, CA, USA). Equal volumes of 2x SDS sample buffer (125 mM Tris-HCl (pH 6.8), 4% (*w*/*v*) SDS, 4% (*v*/*v*) 2-mercaptoethanol, and 20% (*v*/*v*) glycerol)) were added to the lysates, and equivalent amounts of protein (20 *μ*g) were loaded onto 8–12% (*w*/*v*) polyacrylamide gels, electrophoresed, and electrophoretically transferred to polyvinylidene fluoride membranes (Millipore, Billerica, MA, USA). After blocking with 5% (*w*/*v*) skim milk for 30 min, the target antigens were reacted with primary antibodies, followed by addition of secondary antibodies (horseradish peroxidase-conjugated anti-goat IgG or anti-rabbit IgG). Immunoreactive bands were visualized using an enhanced chemiluminescence kit from Amersham Pharmacia Biotech Inc. (Piscataway, NJ, USA).

### 2.4. RNA Isolation and Quantitative Real-Time Polymerase Chain Reaction (PCR)

Total cellular RNAs were isolated using the RNAiso Plus reagent (Takara Bio Inc., Otsu, Japan) according to the manufacturer's instructions. Briefly, HepG2 cDNA was prepared using avian myeloblastosis virus reverse transcriptase and random 9-mer primers. The cDNA was amplified by qPCR using primer sets specific for human TNF-*α*: TGA AAG CAT GAT CCG GGA CG (forward (F)) and TGA GGA ACA AGC ACC GCC TG (reverse (R)); human IL-6: TGT GTG GGG CGG CTA CAT CT (F) and GCC TTC GGT CCA GTT GCC TT (R); and human IL-1*β*: CCT TTG GTC CCT CCC AGG AA (F) and TGA GTC TGC CCA GTT CCC CA (R). Quantitative real-time PCR was performed using SYBR Green Master Mix (Takara Bio Inc.) on a Takara TP-815 instrument. All expression levels were normalized to those of GAPDH.

### 2.5. Immunocytochemistry

Both treated and untreated HepG2 cells were fixed in 4% (*v*/*v*) paraformaldehyde for 15 min, permeabilized for 10 min at room temperature in 0.4% (*v*/*v*) Triton X-100 in PBS, incubated overnight at 4°C with primary antibodies (Santa Cruz Biotechnology Inc., Santa Cruz, CA, USA) (anti-IL-6 (1 : 100), anti-TNF-*α* (1 : 100), and anti-IL-1*β* (1 : 50)), and washed three times with PBS. The VECTASTAIN ABC kit (Vector Laboratories, Burlingame, CA, USA) was used as directed by the manufacturer, followed by addition of 3,3′-diaminobenzidine (DAB). The sections were counterstained with hematoxylin, dehydrated, mounted, and photographed using an ImageScope.

### 2.6. Enzyme-Linked Immunosorbent Assay (ELISA) for IL-6, TNF-*α*, and IL-1*β*

The IL-6, TNF-*α*, and IL-1*β* concentrations in culture supernatants were measured by sandwich ELISA. Monoclonal capture antibodies (4 *μ*g/mL; R&D Systems) were added to 96-well plates (Nunc), and the plates were incubated for 2 hours at room temperature. The plates were incubated with blocking solution consisting of phosphate-buffered saline (PBS) containing 1% BSA and 0.05% Tween 20 for 2 hours at room temperature. The test samples and standard recombinant IL-6, TNF-*α*, and IL-1*β* (R&D Systems) were added to the plates, and the plates were incubated overnight at 4°C. The plates were washed four times with PBS containing Tween 20; then, 200 ng/mL of biotinylated detection monoclonal antibodies (R&D Systems) was added, and the plates were incubated for 2 hours at room temperature. The plates were washed, streptavidin-alkaline-phosphatase (diluted 1 : 2000; Sigma-Aldrich) was added, and the reaction was allowed to proceed for 2 hours at room temperature. The plates were washed four times, and 1 mg/mL of p-nitrophenyl phosphate dissolved in diethanolamine (both from Sigma-Aldrich) was added to induce the color reaction, which was stopped by adding 50 *μ*L of 1 N NaOH. The optical density at 405 nm was measured on an automated microplate reader (Bio-Rad, Hercules, CA, USA). Standard curves were drawn by plotting optical density versus the logs of IL-6, TNF-*α*, and IL-1*β* concentrations.

### 2.7. Statistical Analysis

All experiments were performed two or three times. Data were compared using Student's *t*-test. A *P* value ≤ 0.05 was considered to reflect statistical significance.

## 3. Results

### 3.1. Visfatin Induced Proinflammatory Cytokine Expression in HepG2 Cells

**Figure 1:**
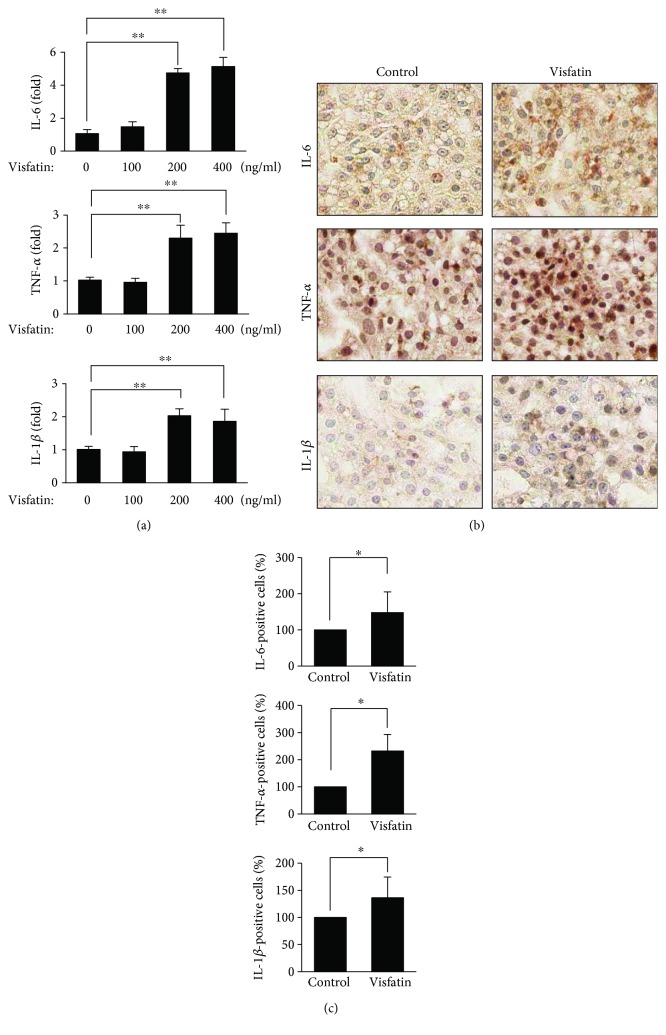
Visfatin induced proinflammatory cytokine synthesis in HepG2 cells. (a) The mRNA levels of inflammatory cytokines (IL-6, TNF-*α*, and IL-1*β*) were measured by real-time PCR. HepG2 cells were treated with different concentrations of visfatin (100–400 ng/mL) for 6 h. The data are means ± standard errors of those of three independent experiments. ^∗∗^*p* < 0.01 compared to the untreated control. (b) HepG2 cells were treated with visfatin at 200 ng/mL for 24 h. The levels of IL-6, TNF-*α*, and IL-1*β* peptides were immunocytochemically assessed. Nuclei were counterstained with hematoxylin. (c) Staining intensities were analyzed using ImageJ software. The basal intensities of cells not exposed to visfatin were set to 100%, and the relative test intensities were then calculated. The data are means ± standard errors of those of three independent experiments. ^∗^*p* < 0.05 compared to the untreated control.

We hypothesized that visfatin might trigger an inflammatory response in hepatocytes. We treated HepG2 cells with visfatin and measured the levels of the proinflammatory cytokines IL-6, TNF-*α*, and IL-1*β*. We used visfatin at concentrations ranging from 100 to 400 ng/mL with reference to previous studies [14, 19]. At 200 and 400 ng/mL, visfatin dramatically increased the mRNA levels of IL-6, TNF-*α*, and IL-1*β* (Figure 1(a)). Next, we immunocytochemically confirmed that the cytokines were produced at the protein level. Visfatin increased expression of IL-6, TNF-*α*, and IL-1*β* (Figure 1(b)). Stained cells were counted in at least 10 zones (100 random cells/zone) and quantified using ImageJ software.

### 3.2. Visfatin Induced Insulin Resistance in a Dose-Dependent Manner in HepG2 Cells

**Figure 2:**
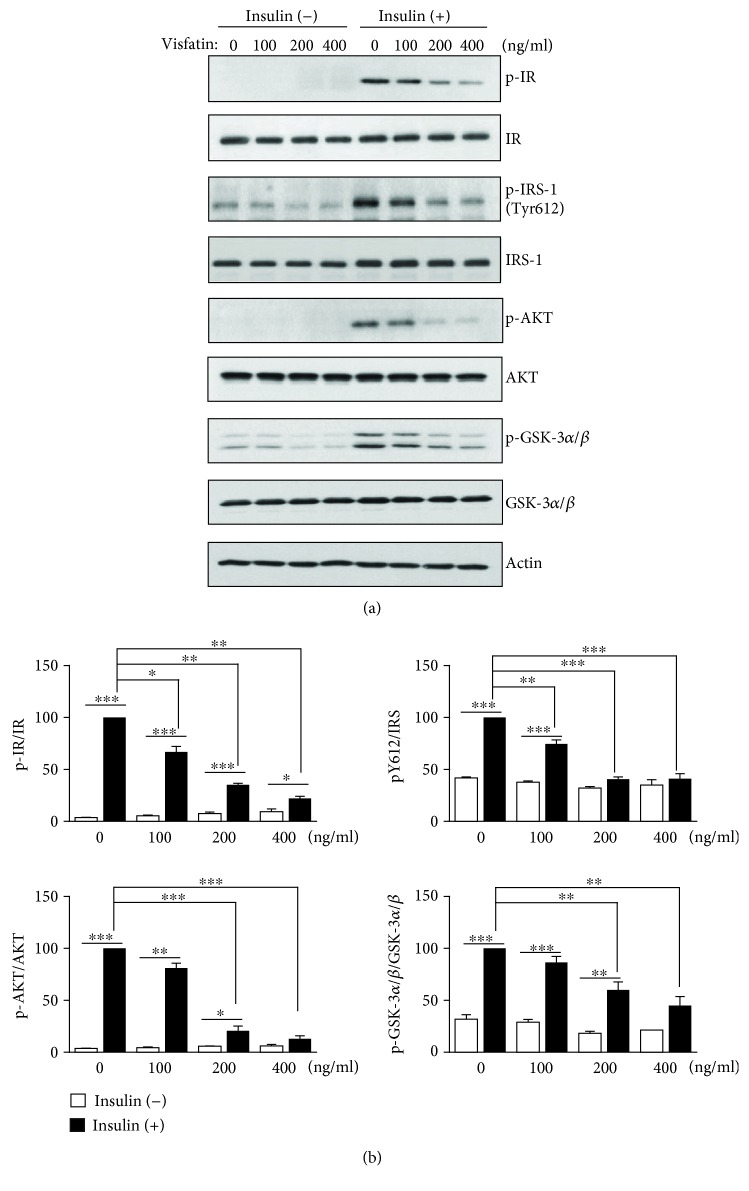
Visfatin impaired insulin signaling. (a) HepG2 cells were treated with different concentrations of visfatin (100–400 ng/mL) for 24 h and then stimulated with 10 nM insulin for 10 min. After harvesting, insulin signaling was analyzed by immunoblotting using antibodies against phospho-IR (p-IR), phospho-IRS-1 (p-IRS-1), phospho-AKT (p-AKT), phospho-GSK-3*α*/*β* (p-GSK-3*α*/*β*), and actin. (b) The phosphoprotein intensities in insulin-treated samples lacking visfatin were set to 100%, and the relative intensities of test samples were then calculated. The data are means ± standard errors of those of three independent experiments. ^∗^*p* < 0.05, ^∗∗^*p* < 0.01, and ^∗∗∗^*p* < 0.001 compared to the insulin-treated control.

We explored the effect of visfatin on the insulin sensitivity of HepG2 cells; insulin signals were measured via immunoblotting using anti-p-IR, anti-p-IRS-1 (Tyr612), anti-p-AKT, and anti-p-GSK-3*α*/*β* antibodies. As shown in Figures 2(a) and 2(b), compared to the control values, visfatin at 100 ng/mL reduced IR phosphorylation by 24% and IRS-1 (Tyr612) phosphorylation by 26% after insulin stimulation. Compared to the control values, visfatin at 200 ng/mL reduced IR phosphorylation by 61%, IRS-1 (Tyr612) phosphorylation by 60%, AKT phosphorylation by 76%, and GSK-3*α*/*β* phosphorylation by 43% after insulin stimulation. Compared to the control values, visfatin at 400 ng/mL decreased IR phosphorylation by 75%, IRS-1 (Tyr612) phosphorylation by 62%, AKT phosphorylation by 85%, and GSK-3*α*/*β* phosphorylation by 60% after insulin stimulation. Thus, visfatin induced insulin resistance in HepG2 cells.

### 3.3. Visfatin Upregulated NF-*κ*B/STAT3 Signaling and IRS-1 S307 Expression in HepG2 Cells

**Figure 3:**
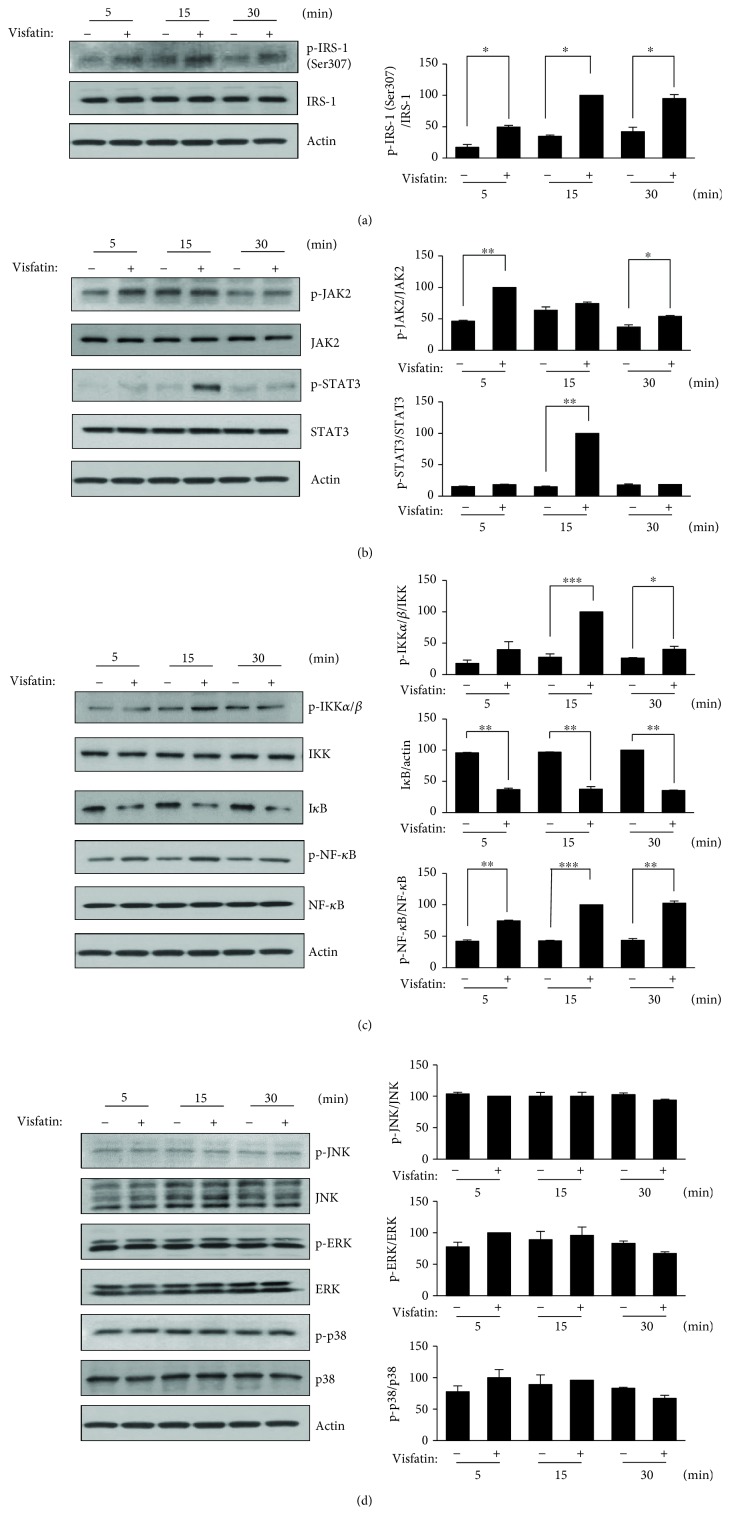
Visfatin activated NF-*κ*B and STAT3 signaling in HepG2 cells. (a) HepG2 cells were incubated with 200 ng/mL visfatin for the indicated times. Inhibitory insulin signaling was analyzed by immunoblotting for phospho-IRS-1 (p-IRS-1, Ser 307) and total IRS-1. ^∗^*p* < 0.05 compared to the untreated control. (b) JAK/STAT3 signaling was analyzed using anti-phospho-JAK2, anti-phospho-STAT3, and anti-actin antibodies. ^∗^*p* < 0.05 and ^∗∗^*p* < 0.01 compared to the untreated control. (c) IKK/NF-*κ*B signaling was analyzed using anti-phospho-IKK*α*/*β*, anti-phospho-NF-*κ*B, I*κ*B*α*, and anti-actin antibodies. ^∗^*p* < 0.05, ^∗∗^*p* < 0.01, and ^∗∗∗^*p* < 0.001 compared to the untreated control. (d) MAP kinase signaling was analyzed using anti-phospho-JNK, anti-phospho-ERK, anti-phospho-p38, and anti-actin antibodies. The maximum control phosphoprotein intensities were set to 100%, and relative test intensities were then calculated. The data are means ± standard errors of those of three independent experiments.

Insulin resistance is associated with increased serine phosphorylation of IRS-1 and inhibition of insulin-stimulated IRS-1 tyrosine phosphorylation and AKT activation [20]. It was reported that serine 307 phosphorylation is activated by the IKK/NF-*κ*B [21] and stress-mediated MAPK pathways [22], and that AKT phosphorylation is decreased by the JAK/STAT pathway [23]. To explore whether visfatin affected phosphorylation of IRS-1 (Ser307), HepG2 cells were treated with visfatin for 5–30 min. Visfatin stimulated IRS-1 (Ser307) phosphorylation in a time-dependent manner but did not change the levels of total IRS-1 or the control protein (*β*-actin) (Figure 3(a)). We used immunoblotting to explore whether visfatin activated the JAK/STAT, IKK/NF-*κ*B, and/or stress-mediated MAPK pathways. Visfatin triggered JAK2 phosphorylation (which was maximal (twofold) at 5 min). STAT3 phosphorylation peaked at 15 min (fivefold) (Figure 3(b)). IKK phosphorylation peaked at 15 min (2.5-fold) and then decreased at 30 min. Visfatin significantly decreased I*κ*B*α* and increased NF-*κ*B phosphorylation, but it did not increase the total NF-*κ*B protein level (Figure 3(c)). Next, we explored whether visfatin activated the MAPK pathway. Visfatin did not affect phosphorylation of JNK, p38, or ERK (Figure 3(d)).

### 3.4. A JAK/STAT3 and an NF-*κ*B Inhibitor Protected against Visfatin-Induced Expression of Inflammatory Cytokines and Insulin Resistance

**Figure 4:**
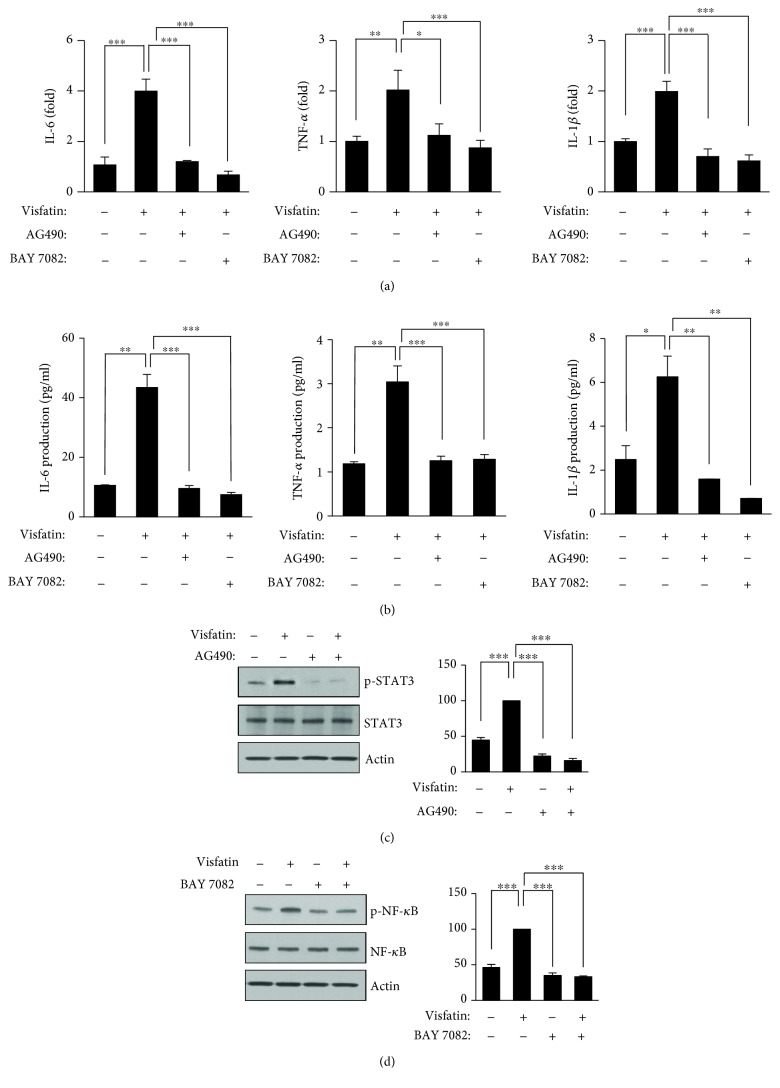
The visfatin-induced increases in proinflammatory cytokine levels are mediated by STAT3 and NF-*κ*B. (a) HepG2 cells were preincubated with AG490 (5 *μ*M) and BAY11-7082 (5 *μ*M) for 1 hour before exposure to visfatin (200 ng/mL) for 6 hours. The levels of the inflammatory cytokines IL-6, TNF-*α*, and IL-1*β* were measured by real-time PCR. (b) HepG2 cells were preincubated with AG490 (5 *μ*M) and BAY11-7082 (5 *μ*M) for 1 hour before exposure to visfatin (200 ng/mL) for 24 hours. The concentrations of IL-6, TNF-*α*, and IL-1*β* in the culture supernatants were measured by ELISA. (c and d) HepG2 cells were preincubated with AG490 (5 *μ*M) and BAY11-7082 (5 *μ*M) for 1 hour before exposure to visfatin (200 ng/mL) for 24 hours. STAT and NF-*κ*B signaling was measured using antibodies against phospho-STAT3, phospho-NF-*κ*B, and actin. The maximum phosphoprotein intensities in visfatin-treated samples were set to 100%, and the relative intensities of test samples were then calculated. The data are means ± standard errors of those of three independent experiments. ^∗^*p* < 0.05, ^∗∗^*p* < 0.01, and ^∗∗∗^*p* < 0.001 compared to the control.

**Figure 5:**
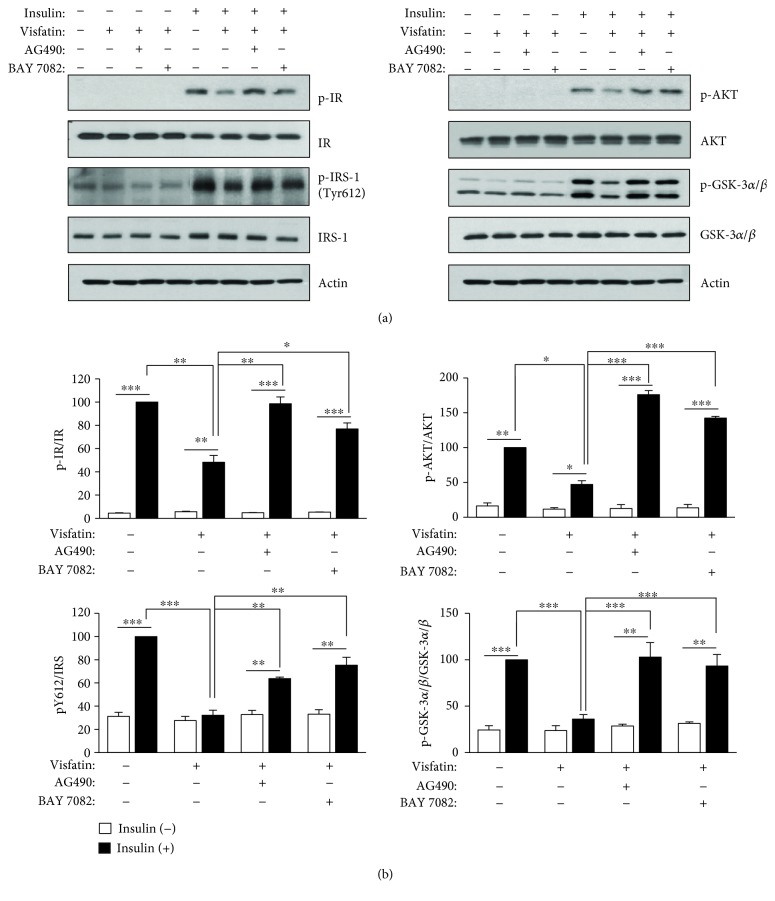
A JAK2/STAT3 inhibitor, AG490, and an NF-*κ*B inhibitor, BAY11-7082, rescue visfatin-impaired insulin signaling. HepG2 cells were pretreated with AG490 (5 *μ*M) or BAY11-7082 (5 *μ*M) for 1 h before exposure to visfatin (200 ng/mL) for 24 h and then stimulated with 10 nM insulin for 10 min. (a) After the cells were harvested, insulin signaling was analyzed by immunoblotting using antibodies against phospho-IR (p-IR), phospho-IRS-1 (p-IRS-1), phospho-AKT (p-AKT), phospho-GSK-3*α*/*β* (p-GSK-3*α*/*β*), and actin. (b) The maximum phosphoprotein intensities in insulin-treated samples without visfatin were set to 100%, and the relative intensities of test samples were then calculated. The data are means ± standard errors of those of three independent experiments. ^∗^*p* < 0.05, ^∗∗^*p* < 0.01, and ^∗∗∗^*p* < 0.001 compared to control.

As visfatin stimulated NF-*κ*B and JAK2-STAT3 signaling, we assumed that such signaling might trigger inflammation and insulin resistance. We first examined the effects of a specific inhibitor of JAK2/STAT3, AG490, and a specific NF-*κ*B inhibitor, BAY11-7082, on the visfatin-induced expression of inflammatory cytokines and insulin resistance in HepG2 cells. AG490 and BAY11-7082 efficiently blocked visfatin-induced IL-6, TNF-*α*, and IL-1*β* RNA expression (Figure 4(a)) as well as secreted IL-6, TNF-*α*, and IL-1*β* levels (Figure 4(b)). Visfatin increased STAT3 phosphorylation twofold, but AG490 completely blocked such phosphorylation (Figure 4(c)). Visfatin increased NF-*κ*B phosphorylation; this was blocked by BAY11-7082 (Figure 4(d)). However, the total protein levels of STAT3 and NF-*κ*B did not change. Next, we investigated whether the visfatin-induced increases in STAT3 and NF-*κ*B activities were associated with insulin resistance. Visfatin reduced the levels of insulin-signaling proteins, including p-IR, p-IRS-1 (Tyr612), p-AKT, and p-GSK-3*α*/*β*, triggering insulin resistance. However, AG490 or BAY11-7082 rescued this insulin resistance. AG490 efficiently blocked the visfatin-induced reductions in p-IR, p-IRS-1 (Tyr612), p-AKT, and p-GSK-3*α*/*β* levels. Also, BAY11-7082 blocked the visfatin-induced reductions in p-IRS-1 (Tyr612), p-AKT, and p-GSK-3*α*/*β* (Figures 5(a) and 5(b)).

## 4. Discussion

In the present study, we found that, in HepG2 cells, visfatin significantly increased the expression levels of proinflammatory cytokines IL-6, TNF-*α*, and IL-1*β* and reduced the expression levels of the insulin-signaling pathway proteins phospho-IRS-1 (Tyr612) and phospho-AKT. Visfatin increased STAT3 and NF-*κ*B pathway activities but not JNK, p38, or ERK pathway activities. A STAT3 or an NF-*κ*B inhibitor blocked visfatin-induced proinflammatory cytokine synthesis and rescued insulin signaling.

NAFLD is a complex spectrum of diseases ranging from simple steatosis to NASH and cirrhosis. NAFLD is strongly associated with insulin resistance and is regarded as the liver manifestation of metabolic syndrome [9]. However, the underlying mechanisms remain unclear. Inflammation is also known to be involved in the progress of NAFLD. IL-6 and TNF-*α* have been suggested to play key roles in NAFLD [24], as have several adipocytokines [10]. Adiponectin was reported to improve hepatic insulin resistance and exert anti-inflammatory effects [25]. Leptin augmented inflammatory and fibrogenic responses in a murine model [26].

Several studies have reported associations between circulating visfatin levels and NAFLD. Serum visfatin levels were higher in females with NAFLD than in controls [27]. Another report showed that serum visfatin levels reflected the extent of portal inflammation [2]. However, some studies reported contradictory results [11]. Moreover, the role, if any, of visfatin in NAFLD development and the mechanisms involved have been rarely studied; we evaluated the effects of visfatin on hepatic insulin resistance and inflammation using HepG2 cells.

It was initially suggested that visfatin acts as an insulin mimic by binding to the insulin receptor (IR) to induce phosphorylation of IRS-1, IRS-2, AKT, and MAPK. However, this report was later retracted [28]; now, IR is no longer considered to serve as a visfatin receptor. Several human studies found positive relationships between circulating visfatin levels and insulin resistance [29], whereas others did not [30]. Any role for visfatin in insulin receptor signaling remains controversial, although there were reports that visfatin upregulated IR phosphorylation in pancreatic *β*-cells and osteoblasts [31, 32]. When we assessed the effects of visfatin in HepG2 cells, we found that visfatin dose-dependently decreased the levels of phospho-IRS-1 (Tyr612) and phospho-AKT but increased the phospho-IRS-1 (Ser307) level. To our knowledge, this is the first study to show that visfatin reduced insulin signaling in hepatocytes. Such inhibition of insulin signaling by visfatin can contribute to the pathogenesis of NAFLD.

Some studies have suggested that visfatin exerted proinflammatory effects, for example, in macrophages [33] and chondrocytes [34]. We evaluated the direct effects of visfatin on proinflammatory cytokine production in hepatocytes and found that visfatin increased production and secretion of the proinflammatory cytokines IL-6, TNF-*α*, and IL-1*β*. The elevated levels of cytokines released from hepatocytes by visfatin may play a role in hepatic inflammation. Further studies of the interactions of visfatin with various cells in the liver and its role in the complex inflammatory process in the development of hepatic inflammation are required.

We explored the effects of visfatin on the signaling pathways involved in insulin signaling and inflammation in hepatocytes. Previous studies reported that visfatin activated the NF-*κ*B pathway in macrophages, endothelial cells, and human vascular smooth muscle cells (VSMCs) [33, 35, 36]. In the vascular endothelium, visfatin causes endothelial dysfunction by increasing inflammatory cytokine production through the NF-*κ*B pathway [35]. Visfatin upregulated inducible nitric oxide synthase synthesis by activating the ERK and the NF-*κ*B of human VSMCs [36]. NF-*κ*B activation via IKK*β*/I*κ*B*α* signaling was involved in the visfatin-induced migration of osteosarcoma cells and IL-6 upregulation [17]. Fan et al. found that visfatin enhanced atheroma inflammation via the NAMPT-MAPK-NF-*κ*B pathway [33]. Here, we found that NF-*κ*B signaling was involved in visfatin-induced inflammation and insulin signaling in hepatocytes. Visfatin enhanced IKK-*β* and I*κ*B*α* phosphorylation; IKK-*β* inhibition abolished visfatin-induced IL-6, TNF-*α*, and IL-1*β* expression and rescued the visfatin-induced inhibition of insulin signaling involving phospho-AKT, phosph-IRS-1 (Tyr612), and GSK-3*α*/*β*.

Visfatin was reported to enhance endothelial IL-6 production and angiogenesis via STAT3 activation [37]. As one of the STAT transcriptional factors activated by JAK, which is itself activated by IL-6 [38], STAT3 serves as both a mediator and a biomarker of endothelial activation. IL-6 triggered hepatic insulin resistance attributable to the “mammalian target of rapamycin” in a manner involving the STAT3-SOCS3 pathway [39]. We found that visfatin increased hepatocyte IL-6 production; thus, we explored whether the JAK-STAT3 pathway was involved in visfatin-induced hepatic production of inflammatory cytokines. Addition of a STAT3 inhibitor followed by visfatin reduced IL-6, TNF-*α*, and IL-1*β* production, suggesting that visfatin exerted proinflammatory effects on hepatocytes via the STAT3 pathway. The JAK-STAT3 pathway was also involved in hepatic insulin signaling. When we treated hepatocytes with visfatin and a STAT inhibitor, the visfatin-induced deterioration of insulin signaling was rescued.

We found that the hepatocyte levels of phospho-JNK, phosphor-p38, and phosphor-ERK were not changed by visfatin, although previous studies reported that visfatin induced p38 and ERK activation in macrophages, enhancing endothelial angiogenesis via the MAPK pathway [40]. The JNK, p38, and ERK pathways did not seem to be involved in the visfatin-induced inflammation and insulin resistance of hepatocytes; the STAT3 and NF-*κ*B pathways appear to be the main pathways in play.

## 5. Conclusion

Our study showed that visfatin induced proinflammatory cytokine production and inhibited insulin signaling via the STAT3 and NF-*κ*B pathways in HepG2 cells. Further studies are needed to determine whether the insulin resistance and inflammation in hepatocytes induced by visfatin play roles in the development of NAFLD.

## Data Availability

The data used to support the findings of this study are available from the corresponding author upon request.

## References

[1] Dahl T. B., Holm S., Aukrust P., Halvorsen B. (2012). Visfatin/NAMPT: a multifaceted molecule with diverse roles in physiology and pathophysiology. *Annual Review of Nutrition*.

[2] Aller R., de Luis D. A., Izaola O. (2009). Influence of visfatin on histopathological changes of non-alcoholic fatty liver disease. *Digestive Diseases and Sciences*.

[3] Samal B., Sun Y., Stearns G., Xie C., Suggs S., McNiece I. (1994). Cloning and characterization of the cDNA encoding a novel human pre-B-cell colony-enhancing factor. *Molecular and Cellular Biology*.

[4] Revollo J. R., Korner A., Mills K. F. (2007). Nampt/PBEF/Visfatin regulates insulin secretion in beta cells as a systemic NAD biosynthetic enzyme. *Cell Metabolism*.

[5] Stastny J., Bienertova-Vasku J., Vasku A. (2012). Visfatin and its role in obesity development. *Diabetes & Metabolic Syndrome: Clinical Research & Reviews*.

[6] Nourbakhsh M., Nourbakhsh M., Gholinejad Z., Razzaghy-Azar M. (2015). Visfatin in obese children and adolescents and its association with insulin resistance and metabolic syndrome. *Scandinavian Journal of Clinical and Laboratory Investigation*.

[7] Nakeeb S. M. S. E., El-Mougy H. M. T., Fatah W. M. E. D. A. E., Bayoumy E. S. M., ElAziz R. E. M. A., Wakeil M. E. S. E. (2014). Serum visfatin in type II diabetes mellitus and its implication in development of diabetic complications. *American Journal of Medicine and Medical Sciences*.

[8] Adams L. A., Lymp J. F., Sauver J. St. (2005). The natural history of nonalcoholic fatty liver disease: a population-based cohort study. *Gastroenterology*.

[9] Tilg H. (2010). Editorial [Hot topic: Adipocytokines in Nonalcoholic Fatty Liver Disease: Key Players Regulating Steatosis, Inflammation and Fibrosis (Executive Editor: Herbert Tilg)]. *Current Pharmaceutical Design*.

[10] Procaccini C., Galgani M., de Rosa V. (2010). Leptin: the prototypic adipocytokine and its role in NAFLD. *Current Pharmaceutical Design*.

[11] Genc H., Dogru T., Kara M. (2013). Association of plasma visfatin with hepatic and systemic inflammation in nonalcoholic fatty liver disease. *Annals of Hepatology*.

[12] Lock J. F., Sponholz C., Hoppe S. (2018). Visfatin/NAMPT is unrelated to nonalcoholic fatty liver histology but correlates with liver recovery after partial liver resection: data from a pilot study. *Integrative Molecular Medicine*.

[13] Mousavi Z., Ganji A., Farrokh Tehrani D., Bahari A., EsmaeilZadeh A., Delghandi M. (2017). Correlation of visfatin level with non-alcoholic fatty liver in metabolic syndrome. *Medical Journal of the Islamic Republic of Iran*.

[14] Choi Y. J., Choi S. E., Ha E. S. (2014). Extracellular visfatin activates gluconeogenesis in HepG2 cells through the classical PKA/CREB-dependent pathway. *Hormone and Metabolic Research*.

[15] Moschen A. R., Kaser A., Enrich B. (2007). Visfatin, an adipocytokine with proinflammatory and immunomodulating properties. *Journal of Immunology*.

[16] Heinrich P. C., Behrmann I., Haan S., Hermanns H. M., Muller-Newen G., Schaper F. (2003). Principles of interleukin (IL)-6-type cytokine signalling and its regulation. *Biochemical Journal*.

[17] Wang G.-J., Shen N.-J., Cheng L., Yehan Fang, Huang H., Li K. H. (2016). Visfatin triggers the in vitro migration of osteosarcoma cells via activation of NF-*κ*B/IL-6 signals. *European Journal of Pharmacology*.

[18] Hotamisligil G. S. (2003). Inflammatory pathways and insulin action. *International Journal of Obesity and Related Metabolic Disorders*.

[19] Yun M. R., Seo J. M., Park H. Y. (2014). Visfatin contributes to the differentiation of monocytes into macrophages through the differential regulation of inflammatory cytokines in THP-1 cells. *Cellular Signalling*.

[20] Rehman K., Akash M. S. H. (2016). Mechanisms of inflammatory responses and development of insulin resistance: how are they interlinked?. *Journal of Biomedical Science*.

[21] Gao Z., Hwang D., Bataille F. (2002). Serine phosphorylation of insulin receptor substrate 1 by inhibitor *κ*B kinase complex. *Journal of Biological Chemistry*.

[22] Gual P., Gremeaux T., Gonzalez T., Le Marchand-Brustel Y., Tanti J. F. (2003). MAP kinases and mTOR mediate insulin-induced phosphorylation of insulin receptor substrate-1 on serine residues 307, 612 and 632. *Diabetologia*.

[23] Thirone A. C. P., JeBailey L., Bilan P. J., Klip A. (2006). Opposite effect of JAK2 on insulin-dependent activation of mitogen-activated protein kinases and Akt in muscle cells: possible target to ameliorate insulin resistance. *Diabetes*.

[24] Tilg H., Diehl A. M. (2000). Cytokines in alcoholic and nonalcoholic steatohepatitis. *The New England Journal of Medicine*.

[25] Polyzos S. A., Kountouras J., Zavos C., Tsiaousi E. (2010). The role of adiponectin in the pathogenesis and treatment of non-alcoholic fatty liver disease. *Diabetes, Obesity & Metabolism*.

[26] Ikejima K., Honda H., Yoshikawa M. (2001). Leptin augments inflammatory and profibrogenic responses in the murine liver induced by hepatotoxic chemicals. *Hepatology*.

[27] Auguet T., Terra X., Porras J. A. (2013). Plasma visfatin levels and gene expression in morbidly obese women with associated fatty liver disease. *Clinical Biochemistry*.

[28] Fukuhara A., Matsuda M., Nishizawa M. (2005). Visfatin: a protein secreted by visceral fat that mimics the effects of insulin. *Science*.

[29] Chang Y. H., Chang D. M., Lin K. C., Shin S. J., Lee Y. J. (2011). Visfatin in overweight/obesity, type 2 diabetes mellitus, insulin resistance, metabolic syndrome and cardiovascular diseases: a meta-analysis and systemic review. *Diabetes/Metabolism Research and Reviews*.

[30] Oki K., Yamane K., Kamei N., Nojima H., Kohno N. (2007). Circulating visfatin level is correlated with inflammation, but not with insulin resistance. *Clinical Endocrinology*.

[31] Brown J. E., Onyango D. J., Ramanjaneya M. (2010). Visfatin regulates insulin secretion, insulin receptor signalling and mRNA expression of diabetes-related genes in mouse pancreatic *β*-cells. *Journal of Molecular Endocrinology*.

[32] Xie H., Tang S. Y., Luo X. H. (2007). Insulin-like effects of visfatin on human osteoblasts. *Calcified Tissue International*.

[33] Fan Y., Meng S., Wang Y., Cao J., Wang C. (2011). Visfatin/PBEF/Nampt induces EMMPRIN and MMP-9 production in macrophages via the NAMPT-MAPK (p38, ERK1/2)-NF-*κ*B signaling pathway. *International Journal of Molecular Medicine*.

[34] Sun L., Chen S., Gao H., Ren L., Song G. (2017). Visfatin induces the apoptosis of endothelial progenitor cells via the induction of pro-inflammatory mediators through the NF-*κ*B pathway. *International Journal of Molecular Medicine*.

[35] Lee W. J., Wu C. S., Lin H. (2009). Visfatin-induced expression of inflammatory mediators in human endothelial cells through the NF-*κ*B pathway. *International Journal of Obesity*.

[36] Romacho T., Azcutia V., Vázquez-Bella M. (2009). Extracellular PBEF/NAMPT/visfatin activates pro-inflammatory signalling in human vascular smooth muscle cells through nicotinamide phosphoribosyltransferase activity. *Diabetologia*.

[37] Kim J. Y., Bae Y. H., Bae M. K. (2009). Visfatin through STAT3 activation enhances IL-6 expression that promotes endothelial angiogenesis. *Biochimica et Biophysica Acta (BBA) - Molecular Cell Research*.

[38] Wang S. W., Sun Y. M. (2014). The IL-6/JAK/STAT3 pathway: potential therapeutic strategies in treating colorectal cancer (review). *International Journal of Oncology*.

[39] Kim J. H., Kim J. E., Liu H. Y., Cao W., Chen J. (2008). Regulation of interleukin-6-induced hepatic insulin resistance by mammalian target of rapamycin through the STAT3-SOCS3 pathway. *Journal of Biological Chemistry*.

[40] Adya R., Tan B. K., Punn A., Chen J., Randeva H. S. (2008). Visfatin induces human endothelial VEGF and MMP-2/9 production via MAPK and PI3K/Akt signalling pathways: novel insights into visfatin-induced angiogenesis. *Cardiovascular Research*.

